# Arthroscopic Partial Meniscectomy for a Degenerative Meniscus Tear Is Not Cost Effective Compared With Placebo Surgery: An Economic Evaluation Based on the FIDELITY Trial Data

**DOI:** 10.1097/CORR.0000000000003094

**Published:** 2024-05-07

**Authors:** Roope Kalske, Ali Kiadaliri, Raine Sihvonen, Martin Englund, Aleksandra Turkiewicz, Mika Paavola, Antti Malmivaara, Ari Itälä, Antti Joukainen, Heikki Nurmi, Pirjo Toivonen, Simo Taimela, Teppo L. N. Järvinen, for the FIDELITY (Finnish Degenerative Meniscal Lesion Study) Investigators

**Affiliations:** a Members of the FIDELITY (Finnish Degenerative Meniscal Lesion Study) Investigators are listed in an Appendix at the end of this article.; 1 Finnish Centre for Evidence-Based Orthopaedics (FICEBO), Department of Orthopaedics and Traumatology, University of Helsinki and Helsinki University Hospital, Helsinki, Finland; 2 Clinical Epidemiology Unit, Orthopedics, Department of Clinical Sciences, Lund University, Lund, Sweden; 3 Centre for Health and Social Economics, National Institute for Health and Welfare, Helsinki, Finland; 4 Pihlajalinna Hospital, Turku, Finland; 5 Pihlajalinna Hospital, Kuopio, Finland; 6 Department of Orthopaedics and Traumatology, Central Finland Central Hospital, Jyväskylä, Finland

## Abstract

**Background:**

In patients with a degenerative tear of the medial meniscus, recent meta-analyses and systematic reviews have shown no treatment benefit of arthroscopic partial meniscectomy (APM) over conservative treatment or placebo surgery. Yet, advocates of APM still argue that APM is cost effective. Giving advocates of APM their due, we note that there is evidence from the treatment of other musculoskeletal complaints to suggest that a treatment may prove cost effective even in the absence of improvements in efficacy outcomes, as it may lead to other benefits, such as diminished productivity loss and reduced costs, and so the question of cost effectiveness needs to be answered for APM.

**Questions/purposes:**

(1) Does APM result in lower postoperative costs compared with placebo surgery? (2) Is APM cost-effective compared with placebo surgery?

**Methods:**

One hundred forty-six adults aged 35 to 65 years with knee symptoms consistent with a degenerative medial meniscus tear and no knee osteoarthritis according to the American College of Rheumatology clinical criteria were randomized to APM (n = 70) or placebo surgery (n = 76). In the APM and placebo surgery groups, mean age was 52 ± 7 years and 52 ± 7 years, and 60% (42 of 70) and 62% (47 of 76) of participants were men, respectively. There were no between-group differences in baseline characteristics. In both groups, a standard diagnostic arthroscopy was first performed. Thereafter, in the APM group, the torn meniscus was trimmed to solid meniscus tissue, whereas in the placebo surgery group, APM was carefully mimicked but no resection of meniscal tissue was performed; as such, surgical costs were the same in both arms and were not included in the analyses. All patients received identical postoperative care including a graduated home-based exercise program. At the 2-year follow-up, two patients were lost to follow-up, both in the placebo surgery group. Cost effectiveness over the 2-year trial period was computed as incremental net monetary benefit (INMB) for improvements in quality-adjusted life years (QALY), using both the societal (primary) and healthcare system (secondary) perspectives. To be able to consider APM cost effective, the CEA analysis should yield a positive INMB value. Nonparametric bootstrapping was used to assess uncertainty. Several one-way sensitivity analyses were also performed.

**Results:**

APM did not deliver lower postoperative costs, nor did it convincingly improve quality of life scores when compared with placebo surgery. From a societal perspective, APM was associated with € 971 (95% CI -2013 to 4017) higher costs and 0.015 (95% CI -0.011 to 0.041) improved QALYs over 2-year follow-up compared with placebo surgery. Both differences were statistically inconclusive (a wide 95% CI that crossed the line of no difference). Using the conventional willingness to pay (WTP) threshold of € 35,000 per QALY, APM resulted in a negative INMB of € -460 (95% CI -3757 to 2698). In our analysis, APM would result in a positive INMB only when the WTP threshold rises to about € 65,000 per QALY. The wide 95% CIs suggests uncertain cost effectiveness irrespective of chosen WTP threshold.

**Conclusion:**

The results of this study lend further support to clinical practice guidelines recommending against the use of APM in patients with a degenerative meniscus tear. Given the robustness of existing evidence demonstrating no benefit or cost effectiveness of APM over nonsurgical treatment or placebo surgery, future research is unlikely to alter this conclusion.

*Level of Evidence* Level III, economic analysis.

## Introduction

An arthroscopic partial meniscectomy (APM) is one of the most common orthopaedic operations, with nearly 800,000 procedures performed in the United States alone each year [11]. The theoretical premise for APM is that by removing torn meniscal fragments and trimming the meniscus back to a stable rim, one can alleviate knee pain and other possible symptoms attributed to a meniscal tear. Most APMs are carried out in middle-aged or older individuals with symptomatic degenerative knee disease [3, 11]. Several recent meta-analyses based on randomized controlled trials (RCTs) showed no treatment benefit of APM over nonsurgical treatment or placebo surgery [10, 22, 43, 45]. However, evidence [2] and expert consensus advocacy [1, 6, 23] for continuing the practice of performing APM for individuals with knee pain has also been presented recently. A recent model-based, cost-effectiveness analysis (CEA), based on an open-label trial, found optional APM after an initial physical therapy period to be cost effective compared with physical therapy alone [47]. The findings prompted the authors to suggest that APM can be a high-value treatment option and should remain a covered treatment option [47].

Conducting economic assessments for medical interventions is challenging, given that different therapeutic alternatives may result in distinct impacts on resource utilization or productivity loss, even if their effects on the clinical outcomes are comparable. For instance, graded activity proved more effective than standard care in reducing the number of workdays missed due to low back pain, even though it did not demonstrate statistically significant improvements in functional outcomes or pain reduction compared with standard care [42]. Some may contend that these concerns are especially relevant when evaluating the cost effectiveness of a surgical procedure because of the potential placebo effect of surgery on clinical outcomes, postoperative resource utilization, and productivity loss.

Given the prevailing uncertainty—and even debate—on the value of arthroscopic surgery for treating degenerative knee conditions [7, 12, 17, 18, 23-25, 31, 33], we set out to provide additional, robust evidence by carrying out a prespecified CEA of our randomized, placebo surgery controlled FIDELITY (Finnish Degenerative Meniscal Lesion Study, APM versus placebo surgery) trial [39, 40]. The obvious strength of this study is the placebo surgery–controlled design that enabled us to isolate the effect of the critical therapeutic element of the APM surgery (the resection of the torn meniscus), thus safeguarding against the inherent confounding related to previous CEAs on the cost effectiveness of the APM procedure (open-label studies comparing APM with various nonsurgical treatment modalities), including the possible placebo effect of surgery.

In this study, we therefore asked: (1) Does APM result in lower postoperative costs compared with placebo surgery? (2) Is APM cost effective compared with placebo surgery?

## Patients and Methods

### Study Design

We report the findings of a prespecified (ClinicalTrials.gov, NCT00549172) cost-effectiveness analysis of the FIDELITY (Finnish Degenerative Meniscal Lesion Study) trial [38-40]. Although the design of the FIDELITY trial has been described in detail previously [39, 40], briefly, it is a multicenter, randomized, participant- and outcome assessor–blinded, placebo-surgery controlled efficacy trial. Participants aged 35 to 65 who had knee symptoms for more than 3 months that were consistent with degenerative medial meniscus tear (clinical diagnosis verified on both MRI and arthroscopy) who were unresponsive to conventional conservative treatment were assigned to APM or placebo surgery (diagnostic arthroscopy plus simulated APM) followed by standardized postoperative care. We excluded patients with a trauma-induced onset of symptoms, locked knee (that could not be straightened normally), previous surgical procedure on the affected knee, clinical knee osteoarthritis (OA) according to American College of Rheumatology criteria [5], radiological knee OA (Kellgren-Lawrence Grade > 1) [21], acute (within the previous year) fracture of the affected extremity, decreased knee ROM, knee instability, MRI assessment showing pathology other than degenerative knee disease requiring treatment other than APM, arthroscopic examination revealing pathology other than a degenerative injury to the medial meniscus requiring intervention other than APM. Participants were followed up with at 2, 6, 12, and 24 months after surgery by questionnaires that also included questions about resource use. At the 24-month follow-up, all participants were also clinically examined by an independent orthopaedic surgeon unaware of the treatment allocation.

### Participants

**Fig. 1:**
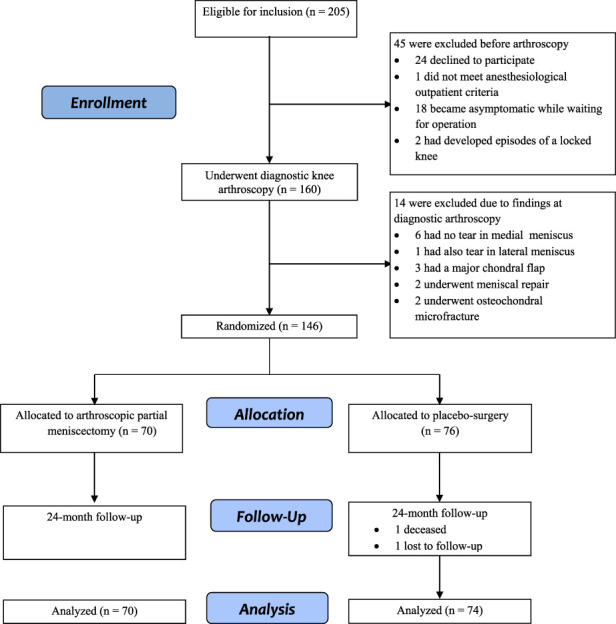
The flow chart of the FIDELITY trial is shown here.

Initially, 205 patients were eligible for enrollment, of whom 12% (24) declined to participate and in total, 22% (45) were excluded. Therefore, 146 patients underwent randomization; 48% (70) were assigned to APM and 52% (76) to placebo surgery. One percent (2 of 146) of patients were excluded from the analyses (both patients were in the placebo surgery group) because of missing data (Fig. 1). Seven percent (5 of 70) of participants in the APM group and 9% (7 of 76) in the placebo-surgery group complained of symptoms severe enough to result in the unblinding of the treatment-group allocation (p = 0.77). All statistical analyses were performed on an intention-to-treat basis. As the frequency of crossover was low, no per-protocol analysis was performed.

### Descriptive Data

In the APM and placebo surgery groups, mean age was 52 ± 7 years and 52 ± 7 years, and 60% (42 of 70) and 62% (47 of 76) of participants were men, respectively. There were no between-group differences in baseline characteristics (Table 1).

**Table 1.** Baseline characteristics of the FIDELITY trial population

Characteristic	APM (n = 70)	Placebo surgery (n = 76)
Mean age in years	52 ± 7	52 ± 7
Men	60 (42)	62 (47)
BMI in kg/m^2^	27 ± 4	28 ± 4
Lysholm knee score	60 ± 15	60 ± 15
WOMET score	56 ± 17	52 ± 18
Knee pain after exercise	6 ± 2	6 ± 2
15D score	0.90 ± 0.06	0.89 ± 0.06

Data presented as mean ± SD or % (n); WOMET (Western Ontario Meniscal Evaluation Tool) and Lysholm scores range from 0 to 100, with 0 indicating the most severe symptoms and 100 the absence of symptoms. Knee pain after exercise (during the preceding week) was assessed on an 11-point numeric rating scale ranging from 0 (no pain) to 10 (extreme pain).

### Patient Involvement

There was no active patient involvement in the design of the study or in the recruitment to or conduct of the study, but patients were involved in the development of one of the main efficacy outcomes (the Western Ontario Meniscal Evaluation Tool) [36].

### Interventions

In both groups, a standard diagnostic arthroscopy was first carried out to rule out the presence of any intra-articular pathology other than a medial meniscus tear. Thereafter, if the patient was randomized to the APM group, the torn meniscus was trimmed to solid meniscus tissue with preservation of as much of meniscus as possible. In the placebo surgery group, APM was mimicked with sounds, sensations, and duration of the operation similar to APM, but without the actual resection of the torn meniscus; as such, surgical costs were the same in both arms and were not included in the analyses. Both groups received standardized postoperative care including a graduated home-based exercise program.

### Costs

In the base case, costs were measured from a societal perspective. The costs included in the CEA arose from the healthcare resource use (including medicine use, primary, and specialized healthcare), the community support (paid home care and caregiving), the loss of productivity because of sickness absence (measured as days), and the travel costs related to all visits to healthcare and community support (Supplemental Table 1; http://links.lww.com/CORR/B296). Costs related to healthcare visits, community support, and loss of productivity were calculated with mean unit costs obtained from the publication *Healthcare Unit Costs in Finland in 2011* [20]. Surgical costs were considered identical between the two treatment groups and thus were not included in our analyses. All prices were converted to 2018 price level euro amounts using Finnish consumer price index 2018 (Statistics Finland, https://www.stat.fi/). In primary analyses, all costs and effects accumulated after the first year were discounted at a rate of 3% following the guidelines of the Ministry of Social Affairs and Health (https://www.finlex.fi/fi/laki/alkup/2009/20090201). Main results are also presented without discounting.

### Outcomes

Our CEA effect measure was change in quality-adjusted life years (QALYs) using the 15D, a generic health-related quality of life (HRQoL) instrument made up of 15 dimensions of health and scored on a scale of 0 (death) to 1 (full health) [41]. 15D utility scores at baseline and the four follow-up measurement points were used to calculate QALYs using the area under the curve assuming a linear relationship between utility values in each measurement point. The generic minimal important difference for the 15D is reported to be +/- 0.015 [4]. We also carried out auxiliary CEAs based on clinical outcome instruments (Supplemental Figs. 1-6 and Supplemental Table 2; http://links.lww.com/CORR/B296).

### Cost-effectiveness Analysis

We conducted a within-trial stochastic CEA over the 2-year FIDELITY trial period by computing the incremental net monetary benefit (INMB) as our primary cost-effectiveness measure. We chose the INMB over the conventionally used incremental cost-effectiveness ratios because the INMB is considered easier to interpret: A treatment (here, APM) is deemed cost effective if the INMB is positive [32]. We calculated the INMB as follows:

INMB = (incremental effects*λ) – incremental costs

where λ represents the willingness to pay (WTP) threshold for one-unit improvement in the outcome (such as, QALY, pain). Although no official WTP thresholds are available in Finland, following previous studies and in line with the threshold applied by NICE (£20,000 to 30,000/QALY), we opted for a value of € 35,000 as the WTP threshold [13, 19, 30]. Incremental effects and costs refer to the mean differences in the outcome and costs between APM and placebo surgery. We estimated the incremental values using seemingly unrelated regression accounting for correlation between costs and effects. In seemingly unrelated regression, two separate regression models (one for outcome and one for costs) were estimated simultaneously assuming their error terms are correlated [29]. We adjusted both models for treatment arm (APM or placebo surgery), age, sex, BMI, absence or presence of minor degenerative changes on a radiograph (Kellgren-Lawrence Grade 0 or 1), study center, and the baseline value of outcome/costs. We performed all analyses according to the intention-to-treat principle and excluded individuals with missing data from the analyses.

We obtained the 95% confidence intervals (CIs) for costs, effects, and INMB using the nonparametric bootstrapping technique with 5000 bootstrap replications by sampling with replacement from the original dataset [9]. The results of the 5000 replications were displayed in cost-effectiveness planes, INMB graphs, and the cost-effectiveness acceptability curves. The INMB graph presents the INMB for different WTP thresholds (€ 0 to € 120,000 here). Cost-effectiveness acceptability curves show the probability of APM being cost effective compared with placebo surgery at various WTP thresholds [14]. Stata v.15 software was used for analyses.

### Ethical Approval

The protocol was approved by the institutional review board of the Pirkanmaa Hospital District (R06157). The study was registered at ClinicalTrials.gov (NCT00549172).

### Sensitivity Analysis

Several one-way sensitivity analyses were conducted to assess the robustness of the results. First, we conducted CEA from a healthcare perspective by excluding travel costs and productivity losses. Second, we assessed the effects of increasing and decreasing productivity losses, hospital costs, and total costs by 20%. Third, we assessed the effects of applying discount rates (on both costs and benefits) of 0% (no discounting) and 6%. Finally, to safeguard against potential bias because of outliers, we also applied a winsorizing procedure (a statistical data preprocessing technique to minimize the effect of extreme values/outliers with replacing them with the observations closest to them) as follows: (1) the top 5% costs were winsorized to the 95th percentile of costs; (2) the bottom and the top 10% costs were winsorized to the 10th and the 90th percentiles, respectively; and (3) the top 5% costs in the APM arm and the bottom 5% costs in the placebo surgery arm were winsorized to the 95th and the 5th percentile, respectively. No power analysis in regard to QALY and costs were performed at the FIDELITY trial planning stage and therefore we cannot provide any estimate on the study power.

## Results

### Does APM Result in Lower Postoperative Costs or Difference in Quality of Life Compared With Placebo Surgery?

APM did not deliver lower postoperative costs, nor did it convincingly improve quality of life scores compared with placebo surgery. The mean discounted total costs were € 7441 ± 10,356 and € 6780 ± 9005 in the APM and placebo surgery arms, respectively (Table 2). Excluding travel costs and productivity losses (that is, applying a healthcare perspective), these figures declined to € 1311 ± 1098 and € 1155 ± 643, respectively. The results of seemingly unrelated regression showed that mean total costs were € 971 (95% CI -2013 to 4017) higher in the APM arm compared with the placebo surgery arm from a societal perspective. The corresponding difference from a healthcare perspective was € 268 (95% CI 37 to 595).

**Table 2.** Mean costs of care in the FIDELITY trial population during 2-year follow-up

Cost category	Costs in €	
	APM (n = 70)	Placebo (n = 74)
Primary care	243 ± 508	170 ± 275
Secondary care	950 ± 536	950 ± 491
Medication	72 ± 207	27 ± 51
Paid help costs	46 ± 222	9 ± 53
Productivity loss	6102 ± 9778	5605 ± 8955
Travel costs	28 ± 57	20 ± 31
Total	7441 ± 10,356	6780 ± 9005

Primary care costs include all costs related to visits at a general practitioner, community health center and occupational health care, and in-patient days at a community health center ward. Secondary care costs include all costs related to all contacts at specialized health care, hospital stays in specialized health care, and MRI imaging.

The patients in the APM and the placebo surgery arms had a mean ± SD discounted QALYs of 1.83 ± 0.10 and 1.80 ± 0.12 years, respectively, over the 2-year follow-up. Seemingly unrelated regression analysis showed that adjusted incremental QALY was 0.015 (95% CI -0.011 to 0.041). The wide confidence intervals suggest a no-difference finding.

### Is APM Cost-effective Compared With Placebo Surgery?

**Fig. 2:**
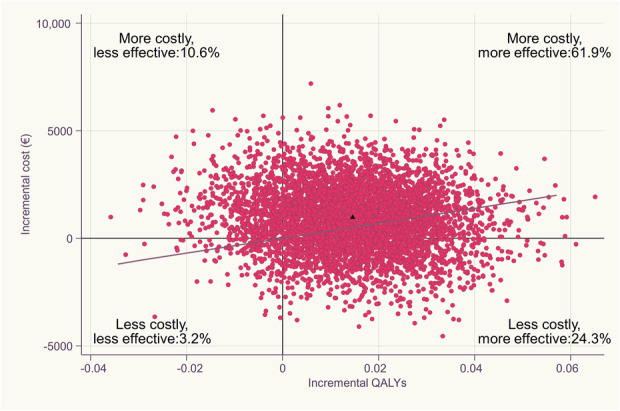
This cost-effectiveness plane of APM versus placebo surgery shows the distribution of bootstrapped estimates and base case result denoted as the triangle. Dashed gray line displays willingness to pay threshold of € 35,000/quality-adjusted life years (QALYs).

**Fig. 3:**
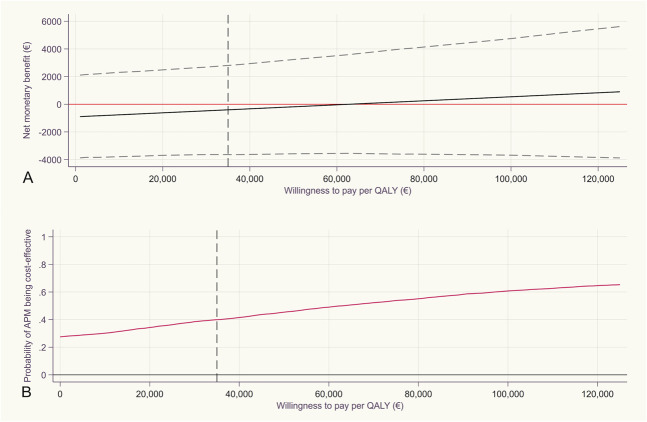
This is a cost-effectiveness acceptability curve and an INMB graph of APM compared with placebo surgery at 2 years postoperatively from the societal perspective. Dashed black vertical lines display willingness to pay threshold of € 35,000/QALYs for the base case analysis.

A favorable outcome in a cost-effectiveness plane (Fig. 2) is typically located in the lower right quadrant, where the studied intervention (here, APM) is more effective and less costly than the comparator (here, placebo surgery). In our study, in less than 25% of bootstrap replications, APM resulted in lower costs and higher QALYs compared with placebo surgery (Fig. 2). To be able to deem APM (or any other medical intervention) cost effective from a societal perspective, the CEA analysis should yield a positive INMB value. Using the conventional WTP threshold of € 35,000 per QALY, APM resulted in a negative INMB of € -460 (95% CI -3757 to 2698) (Fig. 3). The INMB graph (Fig. 3A) revealed that APM would result in a positive INMB only when the WTP threshold rises to about € 65,000 per QALY. Most importantly, the wide 95% CIs suggests uncertain cost effectiveness irrespective of chosen WTP threshold (Fig. 3A). Even at a WTP threshold of € 100,000 per QALY, the probability of APM being cost effective only surpasses 60% (Fig. 3B).

**Fig. 4:**
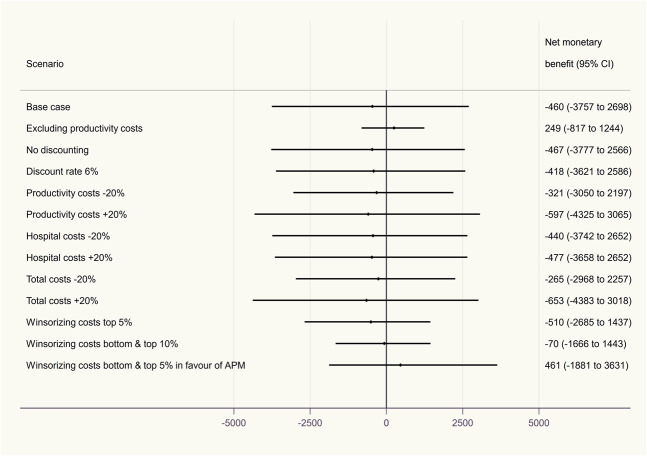
These are the INMBs obtained from a range of one-way sensitivity analyses.

Our conclusion from the base-case analysis was robust to all changes implemented in sensitivity analyses (Fig. 4). The greatest change in INMB was seen when costs were winsorized in favor of APM, but the wide 95% CIs indicate high uncertainty regarding its cost effectiveness.

## Discussion

Despite the robust evidence that shows APM not to offer any clinically relevant benefits over placebo surgery or nonoperative treatment in patients with degenerative meniscus tears, advocates of APM still argue that APM is cost effective. Theoretically, a medical intervention can be proven cost effective even if it does not result in improved clinical outcomes over a comparator, as the cost effectiveness of an intervention compared with another can arise from a difference in treatment effects or a difference in costs associated with treatment options. In our FIDELITY trial–based CEA, we used the follow-up data over a 2-year period to demonstrate that in patients with a degenerative knee disease, a torn meniscus, and no OA according to American College of Rheumatology clinical criteria [5], APM does not result in lower costs nor is it cost effective compared with placebo surgery from either a healthcare payer or a societal perspective. Our findings lend further support to the clinical practice guidelines recommending against performing APM in these patients.

### Limitations

Some limitations are worth discussing. First, the FIDELITY trial was primarily powered to detect a clinically important between-group difference in the primary efficacy outcome measures, and our sample size may leave uncertainty when assessing between-group differences in quality of life and costs. However, the robustness of our findings in the conducted sensitivity analyses suggests it is very unlikely that a larger sample size would have resulted in a different conclusion. Second, it could be argued that the time horizon of 2 years may be too short to elicit the long-term cost effectiveness of APM over placebo surgery. However, given the existing evidence that shows that the possible treatment benefit of APM over nonoperative management is limited in time and absent at 1 to 2 years after surgery, we feel that any beneficial effect of APM beyond our time horizon is very unlikely. By the same token, there is also evidence to suggest that APM may accelerate knee OA progression and may subsequently increase the risk of TKA, inflicting potential increased costs downstream [35, 37, 48]. If anything, the cost effectiveness of APM is likely to diminish—rather than increase—over time. Third, when measuring costs with questionnaires, recall bias can lead to uncertainty in the reported costs. However, this uncertainty should be evenly distributed between study groups and is unlikely to affect our results. Fourth, when basing our healthcare use and community support cost calculations on determined mean unit costs, it is possible that potential individual-level differences in inflicted costs go unnoticed. However, as we have found no differences between the two groups in clinical outcomes and resource use, it is unlikely that these possible differences would change our results.

Generalizability is another concern. Although the clinical results of our trial are likely to be generalizable to other settings, it possible that our cost-effectiveness results do not equally transfer to healthcare systems with different cost structures. Even though it may still not transfer to all healthcare systems, we have addressed this issue by assessing the effects of increasing or decreasing the total costs by 20%, and our results were insensitive to these changes.

Though we consider the placebo surgery controlled design a strength, these trials are occasionally criticized for failing to provide an alternative, viable treatment strategy for the procedure studied. We appreciate this concern; however, we argue that the placebo effect of a surgical procedure may have an effect on the clinical outcomes, the postoperative resource use, and the loss of productivity. Thus, controlling for the placebo effect of a surgical procedure by isolating the critical surgical element (here, the resection of a torn meniscus) as the only difference between the two treatment arms is the only way to assess both the true therapeutic potential and the economic impact of a surgical procedure. Finally, the added value of an economic evaluation can also be questioned in case of no difference in the efficacy, as is the case here [38, 39]. However, there is prior trial evidence from the treatment of musculoskeletal complaints to show that an intervention has been shown to be cost effective despite no benefits in primary (efficacy/effectiveness) outcomes [42]. Furthermore, the CEA methodology elicits possible between-group differences in postoperative resource use and loss of productivity, provides post-APM cost data to inform possible future modeling studies, and mitigates publication bias.

### Discussion of Key Findings

APM neither convincingly improved quality of life nor did it reduce costs of care from the societal perspective when compared with placebo surgery. Endpoints like those seem important for any intervention that patients should undergo or healthcare systems should pay for, and this operation did not deliver them. Several previous studies have reported on comparative costs and the cost effectiveness of APM to nonsurgical treatment with somewhat varying study designs, and perhaps as a consequence, inconsistent conclusions. Three studies, including a retrospective study with no control group and two studies with debatable assumptions in the model-based simulations, concluded that APM might be a cost-effective treatment option [26, 27, 47]. Three other studies—CEAs based on an observational study and on two open-label RCTs—concluded that APM is not an economically attractive treatment option compared with nonoperative treatment [28, 34, 44]. The observational data [34] were limited by confounding by indication and selection bias, whereas a lack of blinding and high rates of crossover (up to 30%) from nonoperative treatment to surgery were reasons for concern in the RCT-based analyses. Of these two RCT-based CEAs, one initially concluded that the total societal costs of physical therapy were lower than those of APM. However, in a secondary CEA of the data, the authors argued that a delayed APM might still be cost effective for nonresponders to physical therapy [46].

Medical interventions that provide little to no clinical benefit in relation to their cost or potential harm to patients are described as forms of low-value care. These interventions are often unnecessary, ineffective, or may even pose risks without corresponding advantages. The low-value care concept has gained prominence in healthcare discussions as part of efforts to improve the efficiency and quality of healthcare delivery. Many of the most common orthopaedic procedures have recently been shown to provide marginal benefits, at best, thus qualifying as low-value care [8, 15, 16]. One might grant that we are facing a high-stakes circumstance where the plausible action would be to severely limit APM surgeries. However, to satisfy orthopaedic surgeons’ concerns regarding the current evidence on APM, and accordingly, to give the advocates of APM their due, we decided to undertake a robust analysis on the cost effectiveness of APM. According to the findings of our CEA, APM appears unlikely to be cost effective regardless of the used WTP threshold. Although this finding may seem obvious since strong evidence suggests APM is clinically ineffective [10, 22, 43, 45], we reiterate that from a health economics aspect, a procedure that produces the same level of effect but at lower costs than a comparator would be a cost effective option. Thus, the potential cost effectiveness of an intervention compared with another needs to be specifically addressed and analyzed. Having said all this, in healthcare settings where APM is still a covered treatment option, we recommend policy makers reassess the evidence base for continuing coverage of APM, as APM appears very unlikely to provide clinical or economic value.

### Conclusion

We found that APM was not cost effective compared with placebo surgery in patients between the ages of 35 and 65 years who had degenerative meniscus tears and no clinical OA. Given the rigorous efficacy design eliminating most biases typically related to studying the efficacy and cost effectiveness of APM, our findings lend further support for clinical practice guidelines that make a strong recommendation against the use of APM in nearly all patients with a degenerative meniscus tear. Considering that APM is associated with a risk of serious adverse events and a proposed increased risk of knee OA progression and future TKA [2, 35, 48], in the prevailing climate of fiscal constraints, APM appears as one of the many medical interventions for which use and justifications should be placed under further scrutiny.

## Group Authors

Members of the FIDELITY (Finnish Degenerative Meniscal Lesion Study) Investigators include: Roope Kalske, Ali Kiadaliri, Raine Sihvonen, Martin Englund, Aleksandra Turkiewicz, Mika Paavola, Antti Malmivaara, Ari Itälä, Antti Joukainen, Heikki Nurmi, Pirjo Toivonen, Simo Taimela, Teppo L.N. Järvinen, Kari Kanto, Timo Järvelä, Janne Lehtinen, Outi Päiväniemi, Marko Raivio, Juha Kalske, Anna Ikonen, Janne Karhunen, Roope Sarvilinna, Sikri Tukiainen, Ville-Valtteri Välimäki, Tero Järvinen, Jani Knifsund, Ville Äärimaa, Tommi Kääriäinen, Heikki Kröger, Janne Sahlman, Jukka Nyrhinen, Juha Paloneva.
